# The Inhibitory Effect of a Novel Polypeptide Fraction from *Arca subcrenata* on Cancer-Related Inflammation in Human Cervical Cancer HeLa Cells

**DOI:** 10.1155/2014/768938

**Published:** 2014-02-09

**Authors:** Yu Wu, Xianjing Hu, Liyan Song, Jianhua Zhu, Rongmin Yu

**Affiliations:** ^1^Biotechnological Institute of Chinese Materia Medica, Jinan University, Guangzhou 510632, China; ^2^Department of Pharmacology, Jinan University, Guangzhou 510632, China

## Abstract

Inflammation is known to be closely associated with the development of cancer. The study was launched in human cervical cancer HeLa cells to investigate the antitumor and anti-inflammatory effects of P2, a marine polypeptide fraction from an important fishery resource *Arca subcrenata*. The basic research showed that P2 could suppress the production of nitric oxide in LPS-induced RAW264.7 macrophage cells as well as the secretion of inflammatory cytokines IL-6 and TNF-**α** in human cervical cancer HeLa cells. For the molecular mechanisms, P2 was shown to downregulate the gene expression of proinflammatory cytokines IL-6 and IL-8 and to inhibit the COX-2 and iNOS-related pathways in HeLa cells. In consequence, P2 might inhibit tumor development by blocking the interaction between tumor microenvironment and proinflammatory mediators. All findings indicate that P2 possesses the potential to be developed as a novel agent for cancer therapy.

## 1. Introduction

In recent years, the studies on the marine natural products, especially marine polypeptides, are attracting more and more attention all over the world. Marine-derived polypeptides with their lower molecular weight and high efficiency have been shown to possess a variety of bioactivities such as antitumor, antiviral, antioxidant [1, 2], anti-inflammatory, and antihyperlipidemic [3] activities and other medicinal properties, and thus they should be considered as a novel source of natural compounds for drug discovery.

The ark shell of *Arca subcrenata*, a bivalve mollusk which lives in the muddy sediment of shallow coastal waters of the north-western Pacific, is a commercially important bivalve species in China, Japan, and Korea [4]. It was reported that the body of *A. subcrenata* could be useful in treatments of tumor, anemia, inflammation, and dyspepsia [5]. A popular traditional Chinese medicine (TCM) called wa leng zi (Concha Arcae) is made of the shell of *A. subcrenata*. Our previous reports testified that *A. subcrenata* evidently improved immunological function *in vivo* [6] and *in vitro* [7, 8], while its hydrolysates exhibited good antioxidant effects, including DPPH radical scavenging and hydrogen peroxide scavenging activities [9].

Inflammation is now well known to have a close relationship with the onset and development of cancer. Epidemiological studies have revealed that chronic inflammation predisposes to different forms of cancer. Cancer of the cervix is the second leading cause of cancer deaths in women worldwide and remains a leading cause of mortality among women of reproductive age in developing countries. Cervical carcinoma arises in women infected with human papilloma virus HPV3 and progresses through a multistage process of carcinogenesis [10], where inflammation plays a crucial role. However, most of the research on *A. subcrenata* polypeptides had focused on antitumor or anti-inflammation, studies about cancer-related inflammation scarcely received attention.

Our preliminary results showed that P2 had the capability of antiproliferation against seven human tumor cell lines, especially for HeLa cell line, which was much more sensitive to P2 than other tumor cell lines [11]. In the current paper, we attempted to explore the effect of P2 with the antitumor property on the proinflammatory cytokines in Hela cells as well as the underlying mechanisms. In order to prove this hypothesis, we investigated firstly the effects of P2 on LPS-induced inflammatory responses in mouse macrophage RAW264.7 cells by Nitric Oxide (NO) Assay Kit. Giving different dosage of P2 toward HeLa cells, we also observed its modulation of the inflammatory response in tumor cells, controlling the release of interleukin-6 (IL-6) and tumor necrosis factor-alpha (TNF-*α*). These results were also supported by reverse-transcription polymerase chain reaction (RT-PCR) analysis and western blot assay on interleukin-8 (IL-8), cyclooxygenase-2 (COX-2) and inducible nitric oxide synthase (iNOS), and so forth.

## 2. Experimental Section

### 2.1. Extraction Procedure and Sample Preparation

*A. subcrenata* was purchased from Huang Sha seafood market (Guangzhou, China). Extraction procedure and sample preparation were the same as those described in our previous paper [11].

### 2.2. Reagents

3-(4,5-Dimethylthiazol-2-yl)-2,5-diphenyltetrazolium bromide (MTT), lipopolysaccharide (LPS), dimethyl sulfoxide (DMSO), penicillin G, and streptomycin were purchased from Sigma Chemical (St. Louis, MO, USA). RPMI-1640 and fetal bovine serum (FBS) were purchased from GIBCO Invitrogen Corporation (San Diego, CA, USA). NO Assay Kit was purchased from Beyotime Institute of Biotechnology (Shanghai, China). Enzyme-linked immunosorbent assay (ELISA) kits for IL-6 and TNF-*α* quantification and GelRed were purchased from R&D Systems (Minneapolis, MN, USA). Prime Script One Step RT-PCR Kit was purchased from Takara BIO Inc. Antibodies against COX-2 and iNOS were purchased from Cell Signaling (Beverly, MA, USA). All other chemicals used were of analytical grade.

### 2.3. Cell Culture and *In Vitro* Cell Viability Assay

The HeLa (human cervical cancer cell) and RAW264.7 cells were obtained from Shanghai Institutes for Biological Sciences, Chinese Academy of Sciences, and were cultured in RPMI-1640 medium, which was supplemented with 10% heat-inactivated FBS, 100 U/mL penicillin, and 100 *μ*g/mL streptomycin in a 5% CO_2_ atmosphere at 37°C. The RAW264.7 cells were cultured in 96-well plates at 5 × 10^5^ cells/mL and treated with P2 or LPS. Phosphate buffered saline (PBS) treatment served as a control. At the end of treatment, 20 *μ*L of a mixture of the tetrazolium compound MTT was added for an additional 4 h at 37°C. The supernatant was aspirated and MTT-formazan crystals were dissolved in 200 *μ*L of DMSO. The absorbance was measured at 570 nm on a microtiter plate reader. Experiments were repeated three times. Results are expressed as a percentage of the survival rate of cells, and values of the PBS-treated group were deducted from the experimental results.

### 2.4. Nitrite Quantification

Nitrite (NO_2_^−^) in the culture medium was determined as described in previous study [12]. The RAW264.7 cells (5 × 10^5^ cells/mL) were stimulated with LPS (1 *μ*g/mL) for 2 h, followed by treatment with various concentrations of P2 for 48 h. The medium was taken, mixed with an equal volume (50 *μ*L) of Griess reagent, and incubated at room temperature for 10 min. Nitrite production was determined by measuring the absorbance at 540 nm. Standard curve was generated with NaNO_2_.

### 2.5. ELISA Analysis

HeLa cells were seeded in 12-well plates at 10^6^ cells/well overnight and then were treated with P2 (1.33, 4, 12 *μ*g/mL, our previous report testified that the IC_50_ for HeLa was 11.43 *μ*g/mL [11]). At the end of incubation, culture supernatants were collected for further analysis. Concentrations of released cytokines IL-6 and TNF-*α* were estimated using human cytokine ELISA kits, as per the manufacturer's instructions.

### 2.6. RT-PCR Analysis

The RT-PCR analysis was used to analyze gene expression of IL-6 and IL-8. HeLa cells were seeded in 6-well plates at 10^6^ cells/well overnight and then were treated with P2 (1.33, 4, 12 *μ*g/mL). RNA was extracted from harvested cells with Trizol reagent (Invitrogen). Conditions of the PCR were initial denaturation at 94°C for 2 min, followed by 30 cycles of the PCR of denaturation at 94°C for 30 s, and annealing at 60°C for 30 s and a final extension at 72°C for 1 min. All primer sequences are shown in Table 1. The housekeeping gene GAPDH was used as an internal control. PCR products were detected on 2% agarose gels, stained with GelRed, and visualized under UV illumination. Semiquantitative analysis was performed using a computerized image analysis system (Image Processing and Analysis in Java).

### 2.7. Western Blotting Analysis

HeLa cells were seeded in 6-well plates at 10^6^ cells/well overnight and then were treated with P2 (1.33, 4, 12 *μ*g/mL). Whole cell lysis was achieved by scraping the cells in lysis buffer (contain 1% PMSF). Lysates were incubated on ice for 30 min before centrifugation (12,000 ×g for 15 min at 4°C), and then the supernatants were collected for further use. Protein concentration of cell lysates was determined by the Bio-Rad protein assay (Bradford assay). For iNOS and COX-2 proteins detection, lysates (15 *μ*L and 25 *μ*L protein, resp.) were separated on 7.5% polyacrylamide-SDS gel for iNOS, or 10% polyacrylamide-SDS gel for COX-2, and transferred to nitrocellulose membranes. The membranes were blocked with 4% BSA for 120 min at room temperature and incubated overnight at 4°C with 1 : 250 or 1 : 1000-diluted specific anti-iNOS or anti-COX-2 antibodies. Thereafter, the membranes were incubated with the appropriate secondary antibody (IgG-HRP conjugates for 120 min at room temperature). Immunoreactivity was detected using enhanced chemiluminescence (ECL) solution followed by exposure to X-ray film. Semiquantitative analysis was performed using a computerized image analysis system (Image Processing and Analysis in Java).

### 2.8. Statistical Analysis

For statistical analyses, a *t*-test was used to compare between two groups. Multiple-group comparisons were evaluated using an analysis of variance (ANOVA) in the SPSS software (Chicago, IL, USA). Differences were defined as significant at  *P* < 0.05.

## 3. Results

### 3.1. Effects of P2 on NO Production in LPS-Stimulated RAW264.7 Cells

**Figure 1:**
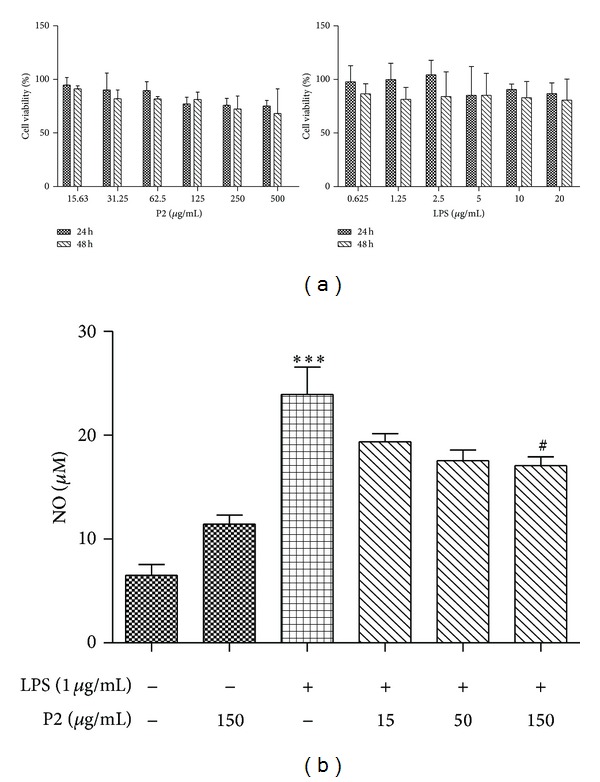
(a) Effects of P2 and LPS on the cell viability of RAW264.7 cells. RAW264.7 cells were treated by P2 and LPS at different concentrations for 24 h and 48 h. Cell viability was determined by MTT assay. The results are representative of three independent experiments and expressed as mean ± SD. Differences were defined as significant at *P* < 0.05. (b) Effects of P2 on NO production in LPS-stimulated RAW264.7 cells. Cells were stimulated with LPS for 2 h followed by treatment with P2 (15–150 *μ*g/mL) for 48 h. Nitrite in the medium was measured using Griess reagent. ***P < 0.001 compared with NC (negative control); ^#^ *P* < 0.05 compared with LPS-treatment only. The results are representative of three independent experiments and expressed as mean ± SD. Differences were defined as significant at *P* < 0.05.

Macrophages play important roles in inflammatory diseases by producing multiple proinflammatory cytokines and enzymes [13] in response to various stimuli including lipopolysaccharide (LPS) and the bacterial endotoxin. NO is a free radical involved in many physiological and pathological processes such as inflammation and tumorigenesis. The production of NO from macrophages plays a key role in inflammation [14]. To observe the effect of P2 on NO production, nitrite, a stable end product of  NO, in the culture media was assayed. Meanwhile, the cell viability was detected. As shown in Figure 1(a), at the concentrations tested, P2 and LPS did not affect cell viability as measured by MTT assay. From Figure 1(b), we can figure out that LPS evoked a fourfold increase in NO production compared with the control. The increase was inhibited by P2 in a dose-dependent manner.

### 3.2. Effects of P2 on Proinflammatory Cytokine Secretion in HeLa Cells

**Figure 2:**
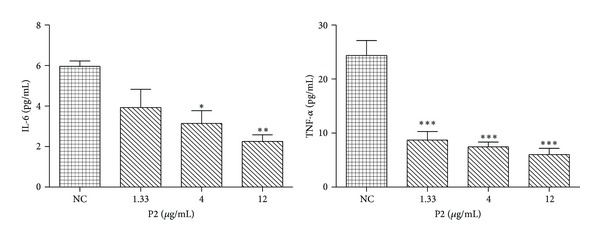
Effect of P2 on proinflammatory cytokine secretion in HeLa cells. Levels of IL-6 and TNF-*α* secreted by HeLa cells after 48 h incubation were assayed by human IL-6 and TNF-*α* ELISA kits. *P < 0.05 and **P < 0.01 compared with normal control (NC). The results are representative of three independent experiments and expressed as mean ± SD. Differences were defined as significant at *P* < 0.05.

Confirming the anti-inflammatory activity of P2 from preliminary experiment, we investigated whether P2 can modulate the secretion of IL-6 and TNF-*α* in HeLa cells. IL-6 is a pleiotropic cytokine that is produced by many different cell types, which plays a role in a wide range of responses. IL-6 increases the metastasis of ovarian cancer cells by effecting their migration and attachment [15]. TNF-*α* mediates a variety of reactions, including immunity, inflammation, and antitumor. TNF-*α* concentrations also tend to be higher in women with cervical carcinoma [16]. In this part, we treated HeLa cells with P2 for 48 h and examined the secretion levels of IL-6 and TNF-*α* by an ELISA assay. At 48 h posttreatment, the IL-6 levels in HeLa cells treated with P2 at 4 and 12 *μ*g/mL decreased by 47.27% and 62.14%, while the TNF-*α* level decreased by 70.45% just at the low concentration (Figure 2). The HeLa cells treated with P2 showed significant attenuation of the cytokine secretion for IL-6 and TNF-*α* in a dose-dependent manner.

### 3.3. Effects of P2 on Proinflammatory Gene Transcription in HeLa Cells

**Figure 3:**
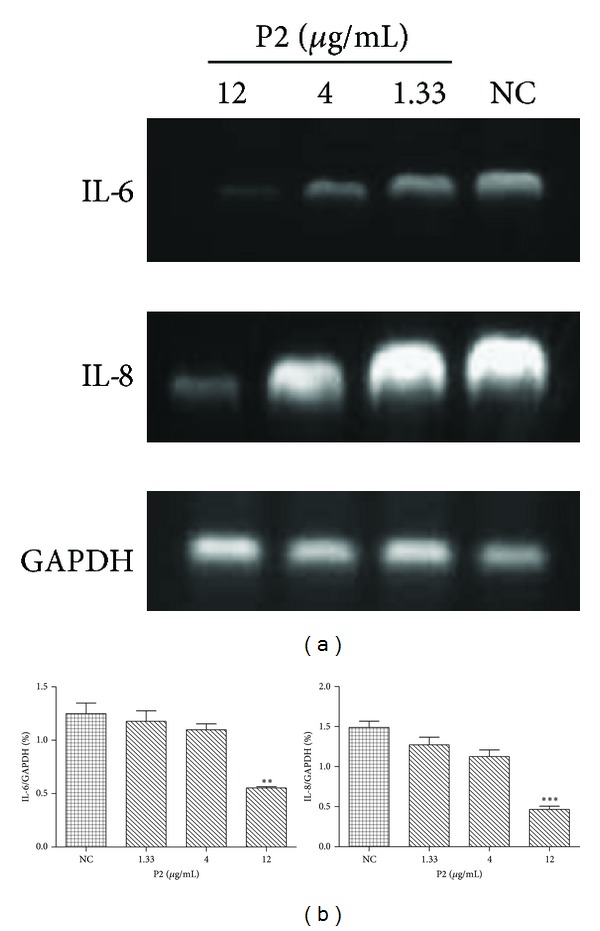
(a) Effect of P2 on immune-related gene expression in HeLa cells. mRNA expression of proinflammatory cytokines in HeLa cells treated with P2 as described in Section 2.6 was analyzed by RT-PCR. GAPDH was used as an internal control. Results are representative of three independent experiments. (b) Quantification of transcript levels by RT-PCR. The primer pair for GAPDH was used as the reference gene. **P < 0.01 and ***P < 0.001 compared with NC. The results are representative of three independent experiments and expressed as mean ± SD. Differences were defined as significant at *P* < 0.05.

To further characterize expression of immune-related genes in HeLa cells, we checked IL-6 and IL-8 expression by RT-PCR after treatment with P2 for 48 h. As shown in Figure 3(a), P2 dose-dependently downregulated the mRNA expression levels of cytokine (IL-6) and chemokine (IL-8) in HeLa cells. The expression levels of IL-6 and IL-8 in HeLa cells treated with P2 (12 *μ*g/mL) were 55.76% and 68.70% lower than the control, respectively (Figure 3(b)).

### 3.4. Effects of P2 on Proinflammatory Protein Expression in HeLa Cells

**Figure 4:**
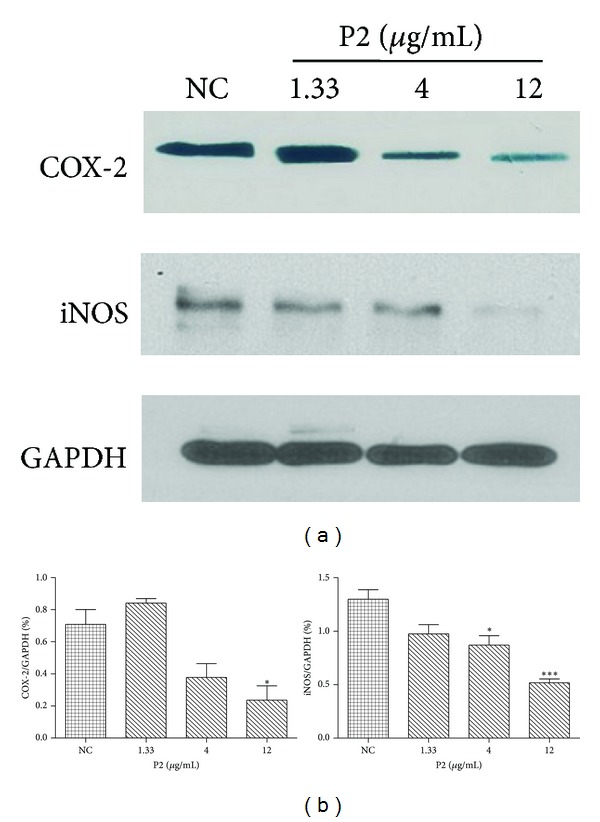
(a) Effects of P2 on immune-related proteins expression in HeLa cells. Following treatment with P2 (1.33, 4, 12 *μ*g/mL), whole lysates were collected and analyzed by western blotting with antibodies specific for COX-2 and iNOS. GAPDH was used as an internal control. Results are representative of three independent experiments. (b) Quantification of expression levels by western blotting. The inhibition of COX-2 and iNOS were 78.67% and 69.65% for the high concentration, respectively, after treatment with P2. GAPDH was used as an internal control. *P < 0.05 and **P < 0.01 compared with NC. The results are representative of three independent experiments and expressed as mean ± SD. Differences were defined as significant at *P* < 0.05.

COX-2 is upregulated in stromal and inflammatory cells by cytokines and other mediators and is expressed constitutively in many human carcinomas [17, 18]. iNOS does not express in normal physiological conditions but in mast cells, macrophages, neutrophils, and various kinds of tumor cells. It plays a critical role in the formation and promotion of tumor through NO synthesis. To understand the underlying mechanism, expression of COX-2 and iNOS in HeLa cells was analyzed. The downregulation of COX-2 and iNOS protein expression was observed within 48 h after the treatment with various concentrations of P2 (1.33–12 *μ*g/mL) as shown by western blot. With the increase of P2 concentrations, the COX-2 and iNOS protein expression was downregulated in a dose-dependent manner (Figure 4). Total protein levels of GAPDH were unaffected. Therefore, we hypothesized that P2 could inhibit proinflammatory protein expression in HeLa cells, which might be related to the COX-2 and iNOS pathways.

## 4. Discussion

Cancer is a multifactorial and multistep disease caused by the accumulation of multiple hits which involves genetic and epigenetic alterations leading to aberrant expression of genes involved in initiation, progression, and promotion of carcinogenesis [19]. How inflammation protects or destroys body tissues is indeed an important issue, particularly in the setting of cancer. Inflammation is not one response but instead represents a dynamic and continuously changing microenvironmental process that has various effects at subsequent stages of tumorigenesis [20]. Cytokines, growth factors, and mediators released in these diseases and the developing tissue microenvironment, such as IL-6, IL-8, TNF-*α*, and NO, have been found to have deleterious properties that pave the way for epithelial mesenchymal transition (EMT), prevent apoptosis, and lead to the destruction of specific host cell-mediated immune responses against tumor antigens. Targeting these factors may decrease the incidence of cancers that develop in the setting of chronic inflammation. Our previous reports indicated that P2 displayed an IC_50_ value below 30 *μ*g/mL *in vitro* and a tumor growth inhibition rate above 30% *in vivo*. In this study, a range of testing results revealed that P2 had a capability of restraining proinflammatory cytokines, so we conjectured that the anti-inflammatory activity of P2 might be binding to the antitumor effect.

Studies on the chemical composition of marine organisms in the last few years have led to the discovery of a variety of organic compounds with known or novel pharmacological and toxic activities on mammalian species. Available evidence suggests that the sea offers a rich source of new organic molecules which may own the unusual structures and the powerful biological activities, used as medicines, biochemical,physiological, or pharmacological tools in biomedical research [21–23]. *A. subcrenata* contains various active ingredients, such as polysaccharides, amino acids, trace elements, vitamins, and polypeptides, which showed good potential antitumor activity [24]. However, no report has demonstrated that *A. subcrenata* polypeptides could inhibit inflammatory response in tumor microenvironment. Cervical cancer is one of the most common malignant cancers in women; and its incidence as well as mortality have an increasing trend in recent years in China. The data from this study demonstrated that P2 could suppress the NO production of LPS-induced RAW264.7 macrophage cells in the experimental dose and keep normal viability (Figure 1). Through further research on human cervical cancer HeLa cells, we could figure out the underlying mechanism.

TNF-*α* appears in the process of inflammation reaction, activating lymphocytes, eosinophils, and diverse inflammatory related factors. It was reported that TNF-*α* would be highly increased in cervicovaginal washings from patients with cervical carcinoma [25]. IL-6 is suggested to provide prognostic value based on its role as a tumor cell growth factor. As shown in Figure 2, P2 at the concentration of 12 *μ*g/mL decreased the IL-6 production by 62.14% in HeLa cells while the TNF-*α* production was reduced by 70.45%.

Inflammation is a host response to a wide variety of tissue injuries characterized by the recruitment of leukocytes from the blood to the injured tissue. This movement is directed by chemokines among which IL-8 plays an important role. Consequently, in the gene level test, we chose IL-6 and IL-8 to conduct the experiment. From Figure 3, we were informed that P2 dose-dependently down-regulated IL-6 and IL-8 mRNAs expression levels in HeLa cells. This result corresponded to that of the above-mentioned study.

There are many cell signaling pathways involved in cancer-related inflammation, including MAPKs, PI3K/AKT, and NF-*κ*B. The transcription factors could start and promote inflammatory gene expression through these pathways, in which NF-*κ*B plays a key role. iNOS, COX-2, IL-6, IL-8, and TNF-*α* secretion by activating NF-*κ*B pathway would cause cell injury, viral infection, inflammation, and tumourigenesis [26, 27]. iNOS and COX-2 are two important kinds of protein in activating NF-*κ*B pathway, and they coordinate with each other significantly inducing the occurrence of cancer [28]. iNOS generates nitric oxide and other toxic nitrogen radicals. In our study, high level of iNOS expression indicated that abundant of NO existed in the untreated HeLa cells. COX-2 is a rate-limiting enzyme involved in the conversion of arachidonic acid to prostaglandins, and it has been identified to have a close relation with tumor genesis [29, 30]. There is abundant documented evidence of elevated expression of COX-2 in cervical tumors and a variety of other malignancies, and the high expression of COX-2 is associated with angiogenesis, lymph node metastasis in cervical cancer [31, 32]. Selective COX-2 inhibitors suppress tumorigenesis in experimental models of colon, breast, prostate, bladder, stomach, skin, and lung cancer [33–38]. As shown in Figure 4, with the increase of P2 concentrations, the protein expression of COX-2 and iNOS was downregulated in a dose-dependent manner comparing with the control. On the basis of the results of this section, it will be important to determine whether P2 could be used as the selective COX-2 inhibitor to treat cervical cancer.

To our knowledge, this is the first report to investigate the anti-inflammatory activity of a novel polypeptide fraction extracted from *A. subcrenata* in human cervical cancer HeLa cells, which is related to its antitumor activity. These tests in this paper support that P2 is capable of downregulating immune-related gene transcription or inhibiting the production of inflammatory cytokines including IL-6, IL-8, and TNF-*α*, as well as the proinflammatory protein expression. In conclusion, our study demonstrates that a novel polypeptide fraction from *A. subcrenata* possesses the inhibitory effect on cancer-related inflammation in human cervical cancer HeLa cells, which provides the scientific foundation for the development of novel antitumor drugs with high efficiency and low toxicity from marine natural products.

## Figures and Tables

**Table 1:** Primers used in this paper.

Gene	Sequence (5′-3′)	Product size (bp)
IL-6	For: CCTGACCCAACCACAAATGC	646
	Rev: CCTTAAAGCTGCGCAGAATGA	
IL-8	For: CTTTCCACCCCAAATTTATCAAAG	264
	Rev: AGAGCTCTCTTCCATCAGAAAGCT	
GAPDH	For: ACACCCACTCCTCCACCTTT	199
	Rev: TAGCCAAATTCGTTGTCATACC	
